# Supplementary figures and images for: Morphological and DNA sequence data uncover a new millipede species in the Thyropygus opinatus subgroup and assign T. peninsularis to this subgroup (Diplopoda: Spirostreptida: Harpagophoridae)
Source: PeerJ. 2025 Jun 6;13:e19277. doi: 10.7717/peerj.19277 (PMC12147765; doi:10.7717/peerj.19277)

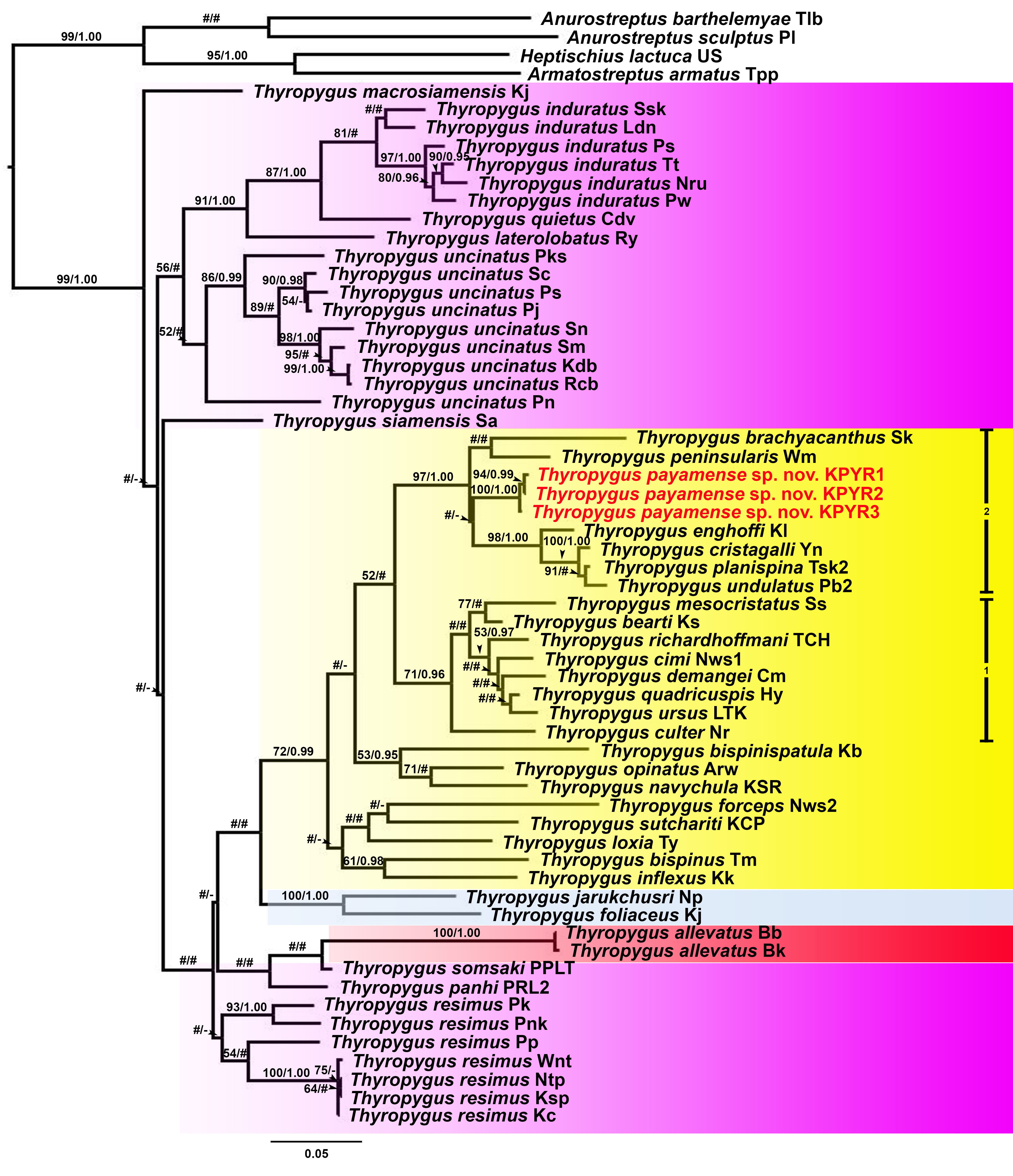

Supplement: Supplemental Information 1 — Numbers at nodes indicate node support based on bootstrapping (ML)/posterior probabilities (BI). Scale bar = 0.05 substitutions/site. # indicates nodes with < 50% bootstrap support and < 0.95 posterior probability. - indicates non - supported nodes. The colored areas mark the T. induratus subgroup (purple), T. cuisinieri subgroup (blue), T. opinatus subgroup (yellow), and T. allevatus subgroup (red). Abbreviations after species names refer to locality names as shown in Table 1. [file peerj-13-19277-s001.jpg]

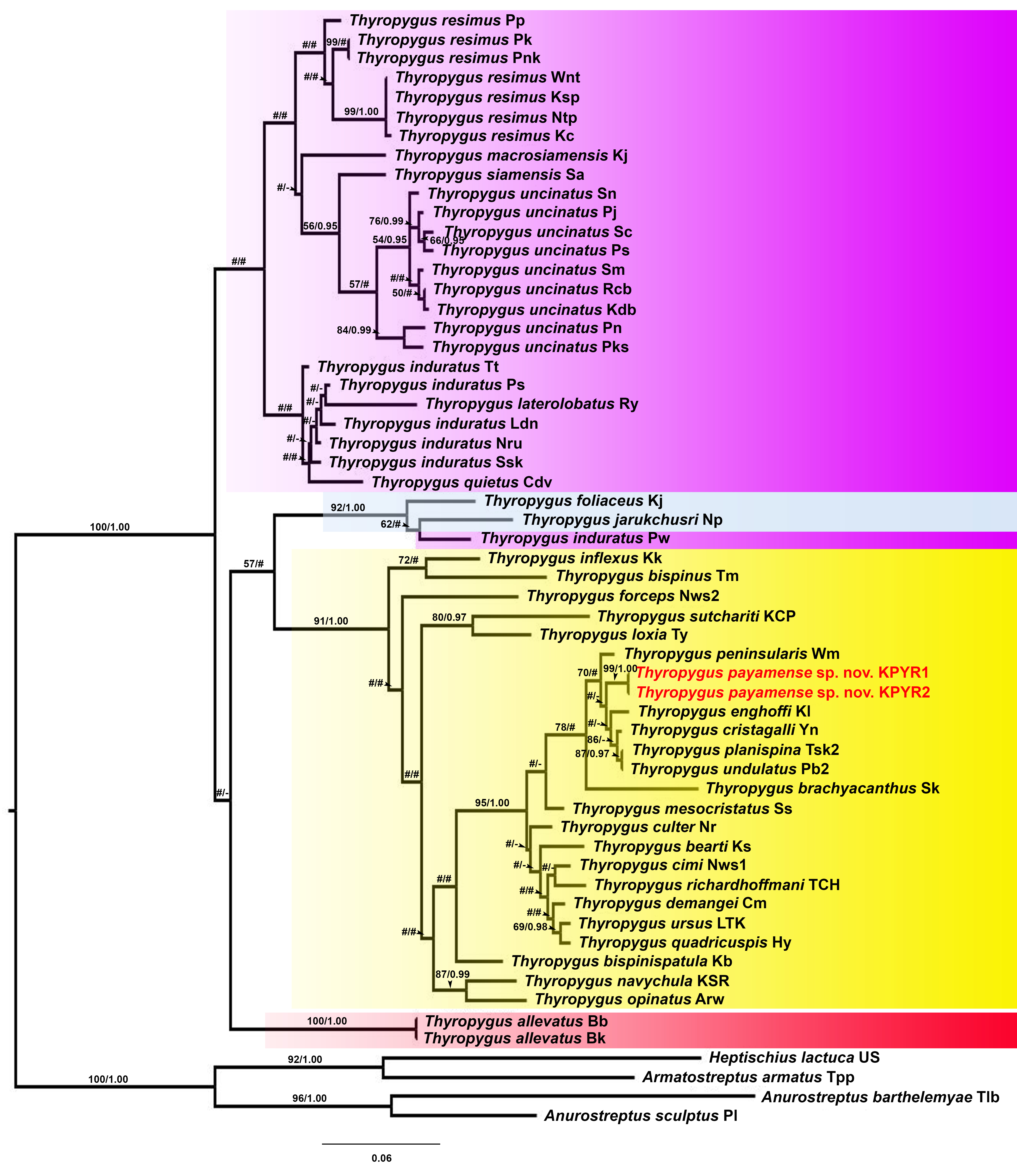

Supplement: Supplemental Information 2 — Numbers at nodes indicate node support based on bootstrapping (ML)/posterior probabilities (BI). Scale bar = 0.05 substitutions/site. # indicates nodes with <50% bootstrap support and <0.95 posterior probability. - indicates non - supported nodes. The colored areas mark the T. induratus subgroup (purple), T. cuisinieri subgroup (blue), T. opinatus subgroup (yellow), and T. allevatus subgroup (red). Abbreviations after species names refer to locality names as shown in Table 1. [file peerj-13-19277-s002.jpg]
