# Supplementary material for: Morphological and DNA sequence data uncover a new millipede species in the Thyropygus opinatus subgroup and assign T. peninsularis to this subgroup (Diplopoda: Spirostreptida: Harpagophoridae)
Source: PeerJ. 2025 Jun 6;13:e19277. doi: 10.7717/peerj.19277 (PMC12147765; doi:10.7717/peerj.19277)
Supplement: Supplemental Information 3 — CUMZ, Museum of Zoology, Chulalongkorn University, Bangkok, Thailand; NHMW, Naturhistorisches Museum, Vienna, Austria. Names of provinces are capitalized. Abbreviations after species names refer to the isolate of each sequence. GenBank accession numbers are indicated for each species. [file peerj-13-19277-s003.docx]

**Table S1.** Specimens from which the COI and 16S rRNA gene fragments were sequenced. CUMZ, Museum of Zool­ogy, Chulalongkorn University, Bangkok, Thailand; NHMW, Naturhistorisches Museum, Vienna, Austria. Names of provinces are in capitals. Abbreviations after species names refer to the isolate of each sequence. GenBank accession numbers are indicated for each species.

|  | **Voucher code** | **Locality** | **COI** | **16S rRNA** |
| --- | --- | --- | --- | --- |
| **Family HarpagophoridaeSubfamily Rhynchoproctinae** |  |  |  |  |
| *Anurostreptus barthelemyae* Demange, 1961 (Tlb) | CUMZ-D00003 | Thale-Ban N.P., Khuan-Don, SATUN, Thailand | KC519469 | KC519543 |
| *A. sculptus* Demange, 1961 (Pl) | CUMZ-D00004 | Phu-Lan-Kha, Nong-Bua- Thale-Ban N.P., Khuan Deang, CHAIYAPHUM, Thailand | KC519470 | KC519544 |
| *Armatostreptus armatus* (Demange, 1983) (Tpp) | CUMZ-D00006 | Tam Pha-Pong temple, Muang, CHIANG MAI, Thailand | KC519472 | KC519546 |
| *Heptischius lactuca* Pimvichai, Enghoff and Panha, 2010 | CUMZ-D00007 | Mae-Usu, Tha-Song-Yang, TAK, Thailand | KC519473 | KC519547 |
| **Subfamily Harpagophorinae** |  |  |  |  |
| ***Thyropygus allevatus* group**  ***T.* *allevatus* subgroup:** |  |  |  |  |
| *T. allevatus* (Karsch, 1881) |  |  |  |  |
| *T. allevatus* Bb | CUMZ-D00013 | Bang Ban, AYUTTHAYA, Thailand | KC519479 | KC519552 |
| *T. allevatus* Bk | CUMZ-D00014 | Bang Kruey, NONTHABURI, Thailand | KC519480 | KC519553 |
|  |  |  |  |  |
| ***T. cuisinieri* subgroup** |  |  |  |  |
| *T. foliaceus* (Demange, 1961) (Kj) | CUMZ-D00050 | Pha Tam, Khong-Chiam, UBONRATCHATHANI, Thailand | KC519514 | KC519589 |
| *T. jarukchusri* Pimvichai, Enghoff and Panha, 2011 (Np) | CUMZ-D00053 | Ban Nong-Pue, Chaturaphakphiman, ROI-ET, Thailand | KC519516 | KC519592 |
|  |  |  |  |  |
| ***T. induratus* subgroup:** |  |  |  |  |
| *T. induratus* Attems, 1936 |  |  |  |  |
| *T. induratus* Ldn | CUMZ-D00022 | Kang Lamduan, Nam-Yeun, UBONRATCHATHANI, Thailand | KC519488 | KC519561 |
| *T. induratus* Nru | CUMZ-D00023 | Nang-Rin waterfall, Non-Sa-Ard, UDONTHANI, Thailand | KC519489 | KC519562 |
| *T. induratus* Ps | CUMZ-D00024 | Phanomsawai, SURIN, Thailand | KC519490 | KC519563 |
| *T. induratus* Pw | CUMZ-D00025 | Phu-Wiang, KHON KAEN, Thailand | KC519491 | KC519564 |
| *T. induratus* Ssk | CUMZ-D00026 | Kantararuk, SRISAKET, Thailand | KC519492 | KC519565 |
| *T. induratus* Tt | CUMZ-D00027 | Ban Ta Toom, Muang, MAHASARAKHAM, Thailand | KC519493 | KC519566 |
| *T. laterolobatus* Pimvichai, Enghoff and Panha, 2011 | CUMZ-D00048 | Koh Sa-Med, Muang, RA-YONG, Thailand | KC519512 | KC519587 |
| *T. macrosiamensis* Pimvichai, Enghoff and Panha, 2011 | CUMZ-D00049 | Pha Tam, Khong-Chiam, UBONRATCHATHANI, Thailand | KC519513 | KC519588 |
| *T. panhai* Pimvichai, Enghoff and Backeljau, 2023 (PRL) | CUMZ-D00151 | Phu Ruea, Phu Ruea, LOEI, Thailand | OP549748 | --- |
| *T. quietus* Attems, 1938 | CUMZ-D00029 | Con Dao N.P., BA RIA-VUNG TAU, Vietnam | KC519494 | KC519568 |
| *T. resimus* Attems, 1938 |  |  |  |  |
| *T. resimus* Kc | CUMZ-D00030 | Kang Ched Khaew, Wat Bod, PHITSANULOK, Thailand | KC519495 | KC519569 |
| *T. resimus* Ksp | CUMZ-D00032 | Kang So-Pa, Nakhonthai, PHITSANULOK, Thailand | KC519496 | KC519571 |
| *T. resimus* Ntp | CUMZ-D00033 | Poi waterfall, Wang-Thong, PHITSANULOK, Thailand | KC519497 | KC519572 |
| *T. resimus* Pk | CUMZ-D00034 | Phu Kum-Khao, Sahussakhan, KALASIN, Thailand | KC519498 | KC519573 |
| *T. resimus* Pnk | CUMZ-D00035 | Phu-No, Ta-Khan-Tho, KALASIN, Thailand | KC519499 | KC519574 |
| *T. resimus* Pp | CUMZ-D00036 | Sae-Ri-Thai cave, Phu-Pan, SAKONNAKHON, Thailand | KC519500 | KC519575 |
| *T. resimus* Wnt | CUMZ-D00037 | Wana-Thara resort, Wang-Thong, PHITSANULOK, Thailand | KC519501 | KC519576 |
| *T. siamensis* Verhoeff, 1938 | CUMZ-D00047 | Chulalongkorn University area, Kang-Khoi, SARABURI, Thailand | KC519511 | KC519586 |
| *T. somsaki* Pimvichai, Enghoff and Backeljau, 2023 (PPLT) | CUMZ-D00152 | Phu Pha Lom, Muang, LOEI, Thailand | OP549749 | --- |
| *T. uncinatus* (Demange, 1961) |  |  |  |  |
| *T. uncinatus* Kdb | CUMZ-D00038 | Khao Kra-Dong, Muang, BURIRAM, Thailand | KC519502 | KC519577 |
| *T. uncinatus* Pj | CUMZ-D00039 | Parchantakham, PRACHINBURI, Thailand | KC519503 | KC519578 |
| *T. uncinatus* Pks | CUMZ-D00040 | Phu-Khae, Chalermprakiat, SARABURI, Thailand | KC519504 | KC519579 |
| *T. uncinatus* Pn | CUMZ-D00041 | Rice field, Ban Nong-Pue, Chaturaphakphiman, ROI-ET, Thailand | KC519505 | KC519580 |
| *T. uncinatus* Ps | CUMZ-D00042 | Phanomsawai, SURIN, Thailand | KC519506 | KC519581 |
| *T. uncinatus* Rcb | CUMZ-D00043 | Rajabhat Buriram, BURIRAM, Thailand | KC519507 | KC519582 |
| *T. uncinatus* Sc | CUMZ-D00044 | Koh Si-Chang, Koh Si-Chang, CHONBURI, Thailand | KC519508 | KC519583 |
| *T. uncinatus* Sm | CUMZ-D00045 | Srimathani Hotel, Muang, NAKHONRATCHASIMA, Thailand | KC519509 | KC519584 |
| *T. uncinatus* Sn | CUMZ-D00046 | Surasammanakhan, Muang, NAKHONRATCHASIMA, Thailand | KC519510 | KC519585 |
|  |  |  |  |  |
| ***T. opinatus* subgroup** |  |  |  |  |
| *T. opinatus* (Karsch, 1881) (Arw) | CUMZ-D00054 | Aerawan waterfall, Srisawad, KANCHANABURI, Thailand | KC519517 | KC519593 |
| *T. inflexus* (Demange, 1989) (Kk) | CUMZ-D00056 | Srinakharin Dam, Srisawad, KANCHANABURI, Thailand | KC519518 | KC519595 |
| *T. bearti* Pimvichai, Enghoff and Panha, 2009 (Ks) | CUMZ-D00057 | Khao Sai, Si Chon, NAKHONSITHAMMARAT, Thailand | KC519519 | KC519596 |
| *T. bispinus* Pimvichai, Enghoff and Panha, 2009 (Tm) | CUMZ-D00058 | Thep Muang Thong temple, Lansak, UTHAITHANI, Thailand | KC519520 | KC519597 |
| *T. bispinispatula* Pimvichai, Enghoff and Panha, 2009 (Kb) | CUMZ-D00059 | Tam Khao Krieb temple, Lang Suan, CHUMPORN, Thailand | KC519521 | KC519598 |
| *T. brachyacanthus* Pimvichai, Enghoff and Panha, 2009 (Sk) | CUMZ-D00060 | Sathitkhirirom temple, Khiriratnikhom, SURATTHANI, Thailand | KC519522 | KC519599 |
| *T. cimi* Pimvichai, Enghoff, Panha and Backeljau, 2016 (Nws1) | CUMZ-D00086 | Namwang Srithammasokarach, Lanska, NAKHONSITHAMMARAT, Thailand | KU306519 | KU306526 |
| *T. cristagalli* Pimvichai, Enghoff and Panha, 2009 (Yn) | CUMZ-D00064 | Koh Yao Noi, Koh Yao, PHANG-NGA, Thailand | KC519523 | KC519603 |
| *T. culter* Pimvichai, Enghoff, Panha and Backeljau, 2016 (Nr) | CUMZ-D00078 | Rorn waterfall, Khlong Thom, KRABI, Thailand | KC519535 | KC519616 |
| *T. demangei* Pimvichai, Enghoff and Panha, 2009 (Cm) | CUMZ-D00066 | Ban Chang Lang, Hat Chao Mai, Si kao, TRANG, Thailand | KC519525 | KC519605 |
| *T. enghoffi* (Demange, 1989) (Kl) | CUMZ-D00067 | Khao Lak Lam Ru, Takuepa, PHANG-NGA, Thailand | KC519526 | KC519606 |
| *T. forceps* Pimvichai, Enghoff, Panha and Backeljau, 2016 (Nws2) | CUMZ-D00073 | Namwang Srithammasokarach, Lanska, NAKHONSITHAMMARAT, Thailand | KC519531 | KC519611 |
| *T. loxia* Pimvichai, Enghoff and Panha, 2009 (Ty) | CUMZ-D00065 | Tam Yai temple, Tha Cha Na, SURATTHANI, Thailand | KC519524 | KC519604 |
| *T. mesocristatus* Pimvichai, Enghoff, Panha and Backeljau, 2016 (Pb2) | CUMZ-D00077 | Srikasorn, Rattaphum, SONGKHLA, Thailand | KC519534 | KC519615 |
| *T. navychula* Pimvichai, Enghoff, Panha and Backeljau, 2016 (KSR) | CUMZ-D00089 | Surin Islands, Khuraburi, PHANG-NGA, Thailand | KU306522 | KU306529 |
| *T. peninsularis* Hoffman, 1982 (Wm) | CUMZ-D00011 | Wang-Matcha, Kapoe, RANONG, Thailand | KC519477 | KC519551 |
| *T. planispina* Pimvichai, Enghoff, Panha and Backeljau, 2016 (Tsk2) | CUMZ-D00088 | Tham Sua temple, Muang, KRABI, Thailand | KU306521 | KU306528 |
| *T. quadricuspis* Pimvichai, Enghoff and Panha, 2009 (Hy) | CUMZ-D00069 | Khao Pina temple, Hui Yod, TRANG, Thailand | KC519528 | KC519608 |
| *T. richardhoffmani* Pimvichai, Enghoff and Panha, 2009 (TCH) | CUMZ-D00085 | Tham Chang Hai, Hui Yod, TRANG, Thailand | KU306518 | KU306525 |
| T. *sutchariti* Pimvichai, Enghoff, Panha and Backeljau, 2016 (KCP) | CUMZ-D00090 | Kaeng Krachan, Kaeng Krachan, PHETCHABURI, Thailand | KU306524 | KU306531 |
| *T. undulatus* Pimvichai, Enghoff, Panha and Backeljau, 2016 (Pb2) | CUMZ-D00087 | Khao Pha Nom Bencha, Muang, KRABI, Thailand | KU306520 | KU306527 |
| *T. ursus* Pimvichai, Enghoff, Panha and Backeljau, 2016 (LTK) | NHMW-Inv.7855 | Lanta Islands, Koh Lanta, KRABI, Thailand | KU306523 | KU306530 |
| *T. payamense* **sp. nov.** KPYR1 | CUMZ-D00155 | Aow Yai, Payam Island, Muang, RANONG, Thailand | PV019345 | PV029246 |
| *T. payamense* **sp. nov.** KPYR2 | CUMZ-D00155-2 | Aow Yai, Payam Island, Muang, RANONG, Thailand | PV019346 | PV029247 |
| *T. payamense* **sp. nov.** KPYR3 | CUMZ-D00155-2-1 | Aow Yai, Payam Island, Muang, RANONG, Thailand | PV019347 | --- |
