# Supplementary material for: Morphological and DNA sequence data uncover a new millipede species in the Thyropygus opinatus subgroup and assign T. peninsularis to this subgroup (Diplopoda: Spirostreptida: Harpagophoridae)
Source: PeerJ. 2025 Jun 6;13:e19277. doi: 10.7717/peerj.19277 (PMC12147765; doi:10.7717/peerj.19277)
Supplement: Supplemental Information 9 [file peerj-13-19277-s009.docx]

**Key to the 29 currently recognized species of the *T. opinatus* subgroup; figures underneat a couplet illustrate the relevant gonopodal characteristics referred to in the couplet** (updated from Pimvichai et al., 2016)

1. Apical part of telopodite with spatulate lobe (*sl*)……………………...……………………....2

− Apical part of telopodite with lamellar lobe (*ll*)……………….………...…………..………22

| 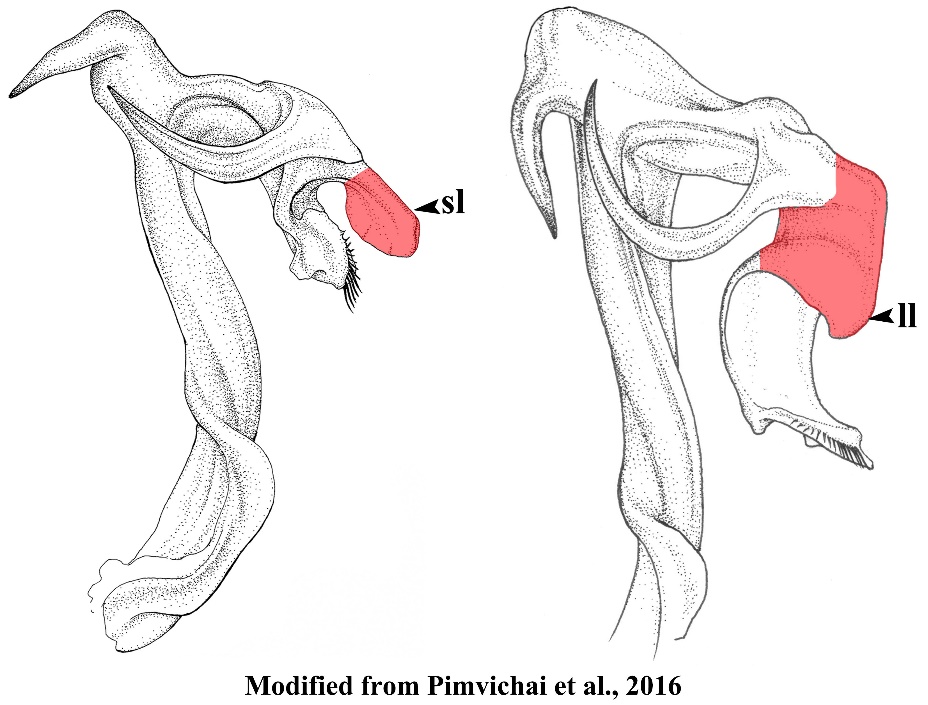 |
| --- |
|  |

2. Spatulate lobe (*sl*) distally drawn out into one or two sharp dark brown spine(s)…….………3

− Spatulate lobe (*sl*) distally expanded and/or rounded, spoon-like, without a spine...…........…9

| 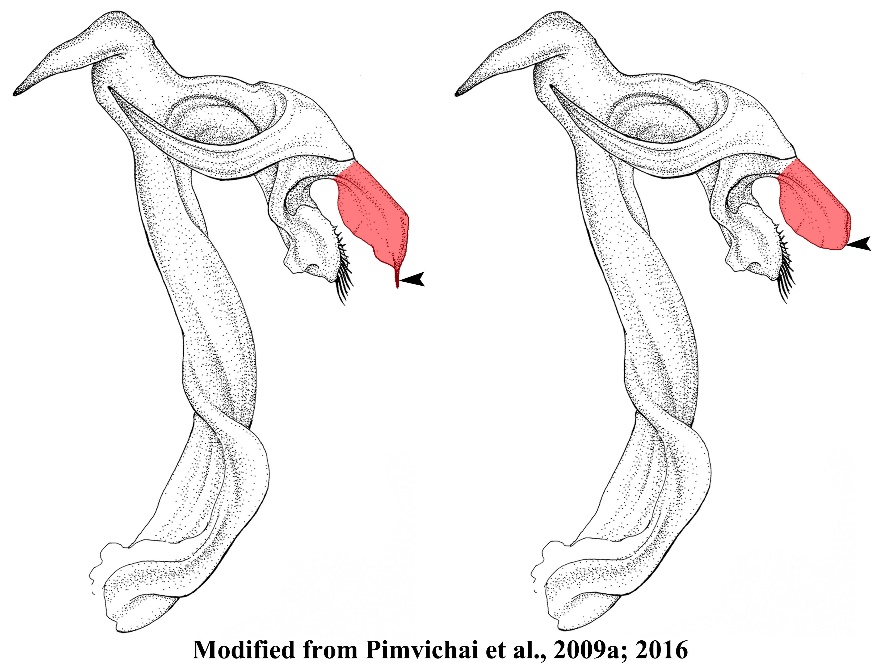 |
| --- |

3. Spatulate lobe (*sl*) terminating in two sharp brown spines, the outer spine slightly smaller and shorter than the inner one; lateral process of anterior coxal fold (*alp*) slender, slightly curving mesad; mesal process of anterior coxal fold (*amp*) almost as long as *alp*, flattened…………... ..………………………….…………………………………………………...***T. bispinispatula***

− Spatulate lobe (*sl*) terminating in a single sharp dark brown spine...........................................4

4. Telopodite without a lobe distal to *fe*; lateral process of anterior coxal fold (*alp*) long, slender, regularly curved, tip close to tip of opposite *alp*, the two together forming a circle; mesal process of anterior coxal fold (*amp*) straight, shorter than *alp*; femoral spine (*fe*) directed distad, pointed……………………………..…………..……………...….....***T.*** ***erectus***

− Telopodite distally to *fe* with a large, round lobe (*lo*) projecting distolaterally…………..…..5

| 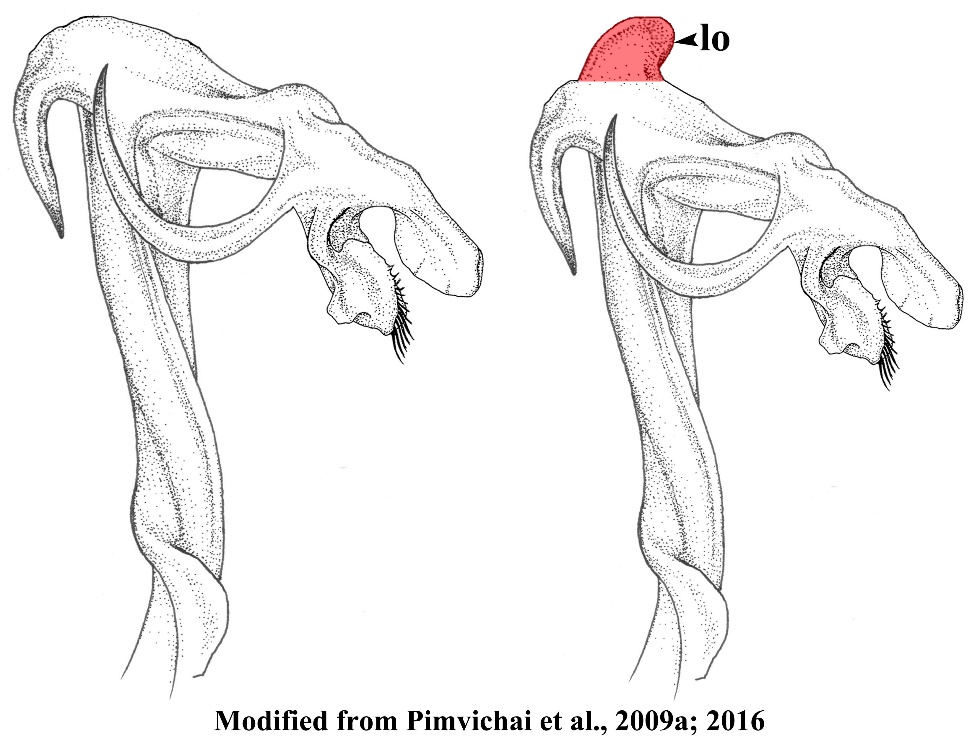 |
| --- |

5. Lateral process of anterior coxal fold (*alp*) very slender, regularly curved………………..….6

− Lateral process of anterior coxal fold (*alp*) different, broader and/or with several apical denticles.....................................................................................................................................8

| 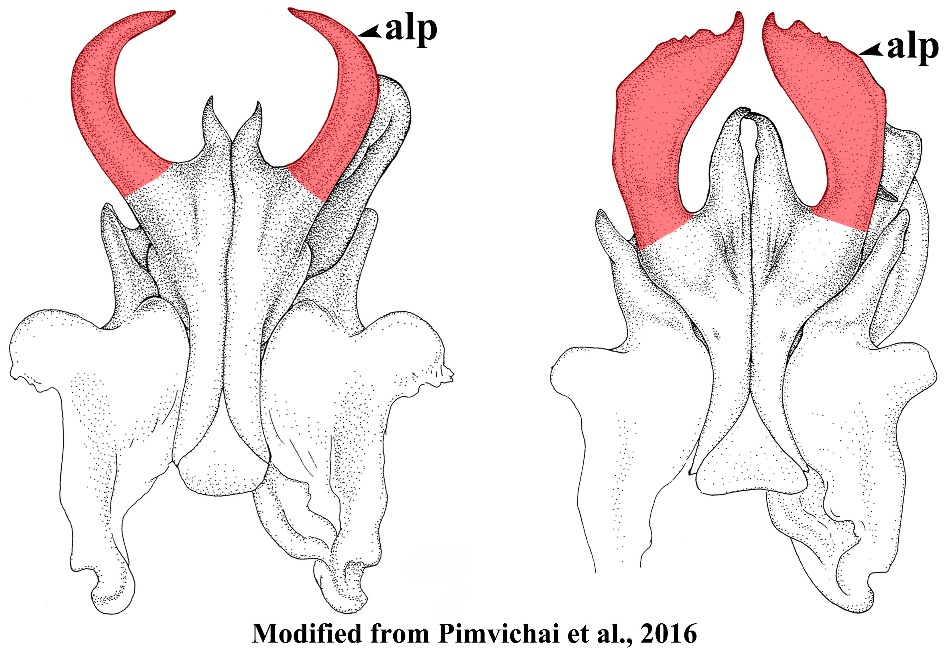 |
| --- |

6. Mesal margin of lateral process of anterior coxal fold (*alp*) with fine serrations; mesal process of anterior coxal fold (*amp*) almost as long as *alp*, broadly expanded, apically sharp, straight distad, mesal margin forming a strong longitudinal crest (*lc*) in posterior view……………………………………...……………………………………….***T. navychula***

− Mesal margin of lateral process of anterior coxal fold (*alp*) without serrations, tip of lateral process close to tip of the opposite side, the two together forming a circle……………….….7

7. Mesal process of posterior coxal fold (*pmp*) strongly developed along anterior-posterior axis...............................................................................................................................***T. floweri***

− Mesal process of posterior coxal fold (*pmp*): slender, directed distolaterad…......….***T. forceps***

8. Lateral process of anterior coxal fold (*alp*) broad, apically gradually narrowed; mesal process of anterior coxal fold (*amp*) almost as long as lateral process (*alp*), slender, straight, terminally slightly curved, pointed...........................................................................***T. opinatus***

− Lateral process of anterior coxal fold (*alp*) apically bent abruptly mesad, tip with serrate margins; mesal process of anterior coxal fold (*amp*) much shorter than lateral process (*alp*), directed mesodistad, simple, pointed; mesal process of posterior coxal fold (*pmp*): strongly developed along anterior-posterior axis………….………...……………..……...***T. implicatus***

9. Telopodite with a single femoral spine (*fe*)…………...………………………....…..………10

− Telopodite with two femoral spines (*fe 1* and *fe 2*)…………………...………..……………19

| 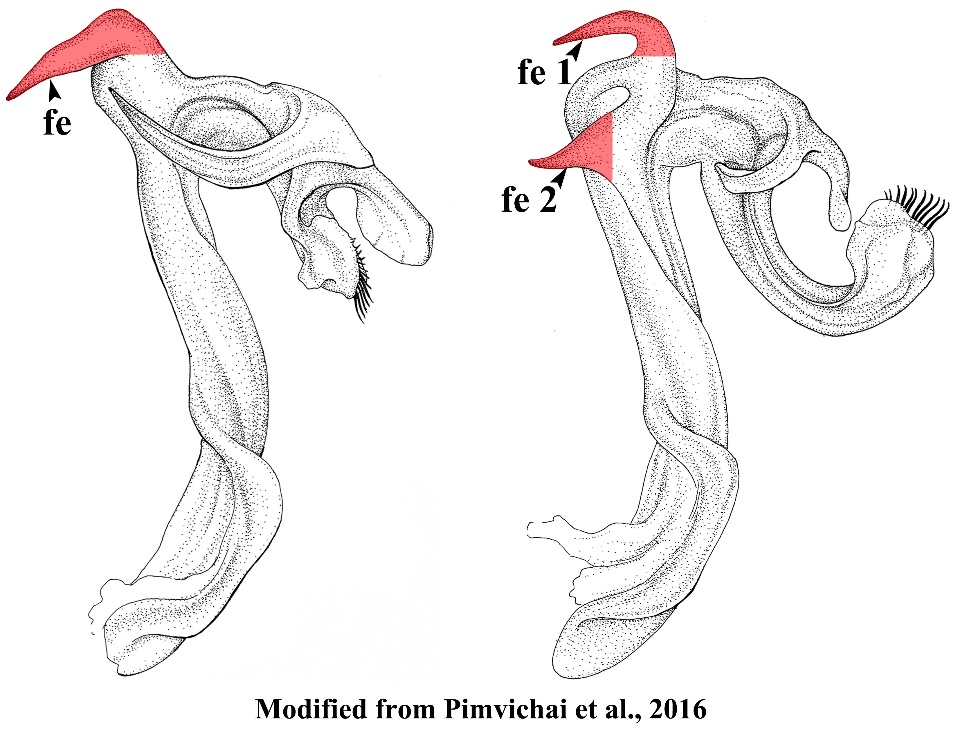 |
| --- |

10. Mesal process of anterior coxal fold (*amp*) short……………………………..……………..11

− Mesal process of anterior coxal fold (*amp*) long, slender………..………..……....................13

| 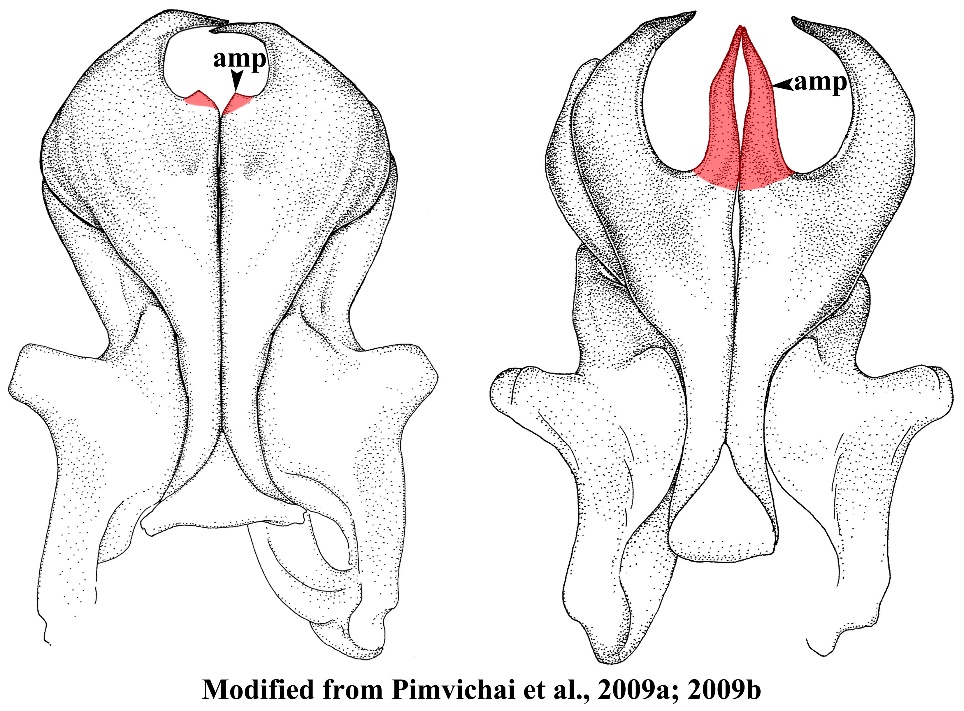 |
| --- |

11. Telopodite with slender tibial spine (*ti*), not curving mesad; *fe* curving backward, without small spine; mesal process of anterior coxal fold (*amp*) very short, pointed......***T. peninsularis***

− Telopodite with short, slender tibial spine (*ti*), curving mesad …………………..………….12

12. Femoral spine (*fe*) with a small, slender, pointed spine (*sfe*) at base (Fig. 3C); mesal process of anterior coxal fold (*amp*) short, forming a triangular process; telopodite distally to *fe* without a small round lobe (*lo*)……………………...……….......….…***T. payamense* sp. nov.**

− Femoral spine (*fe*) without a small slender, pointed spine (*sfe*) at base; telopodite distally to *fe* with a small round lobe (*lo*) projecting distolaterally.....................................................***T. loxia***

13. Lateral process of anterior coxal fold (*alp*) apically abruptly truncate………...…….. ***T. bearti***

− Lateral process of anterior coxal fold (*alp*) apically pointed………………………….……..14

| 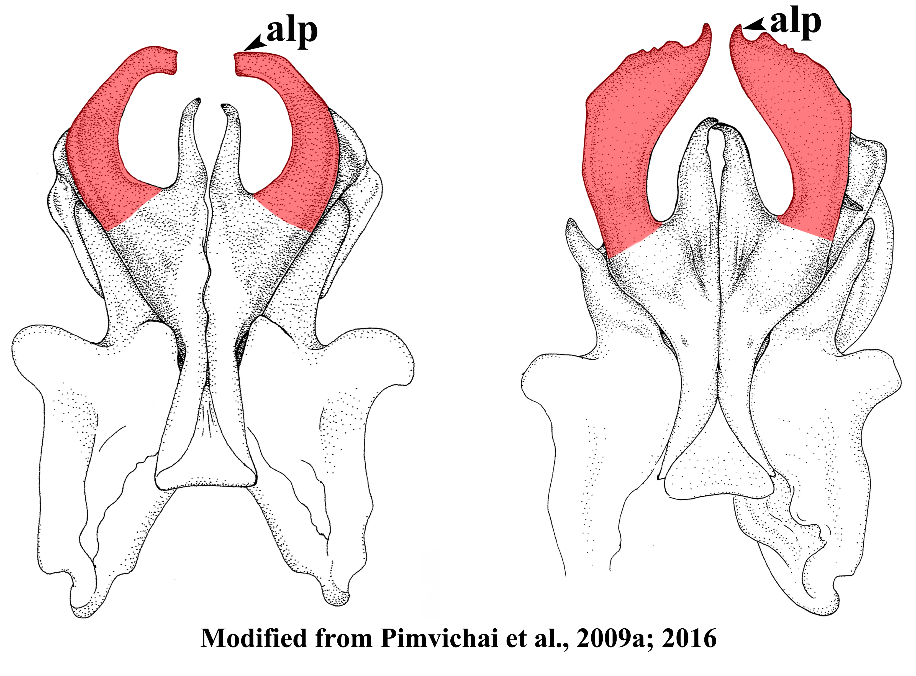 |
| --- |

14. Mesal process of anterior coxal fold (*amp*) shorter than lateral process (*alp*)...…………......15

− Mesal process of anterior coxal fold (*amp*) as long as lateral process (*alp*)…………...….....16

| 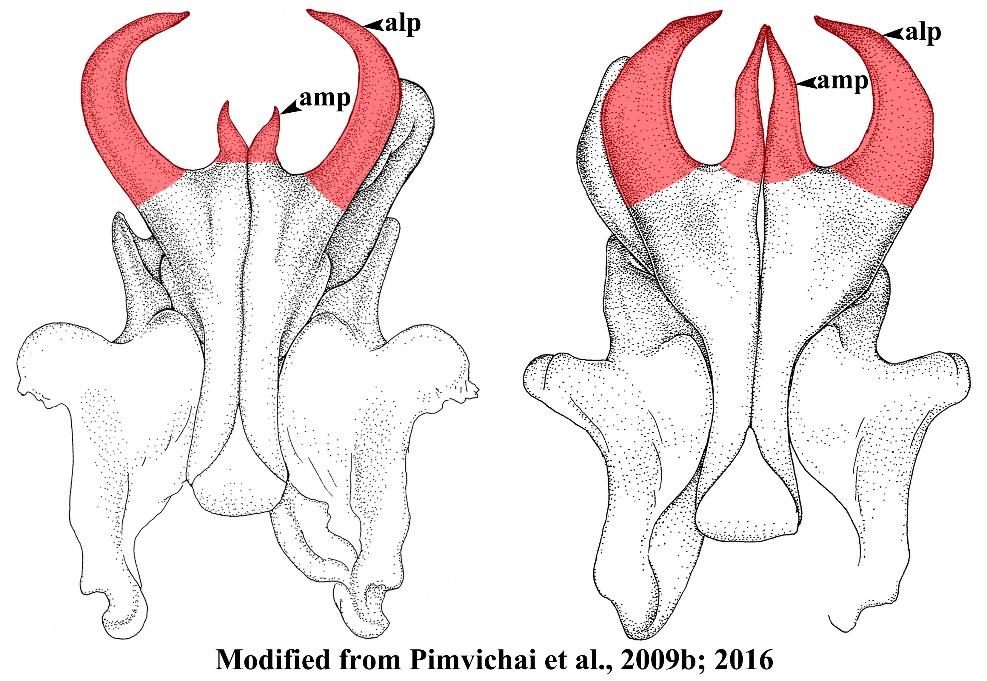 |
| --- |

15. Mesal process of anterior coxal fold (*amp*) directed obliquely distomesad, slender, straight

.*..........................*...................................................................................................*...........****T. chelatus***

− Mesal process of anterior coxal fold (*amp*) directed distad, thicker, slightly sigmoid…………

………………………………………………………………………………*.....****T. brachyacanthus***

16. Mesal process of anterior coxal fold (*amp*) directed obliquely distomesad, tip overlapping tip of opposite *amp*; lateral process of posterior coxal fold (*plp*) a massive, broad lobe, projecting laterad….……………………….……….…………......................……***T. sutchariti***

− Mesal process of anterior coxal fold (*amp*) directed distad…………………....…..………...17

| 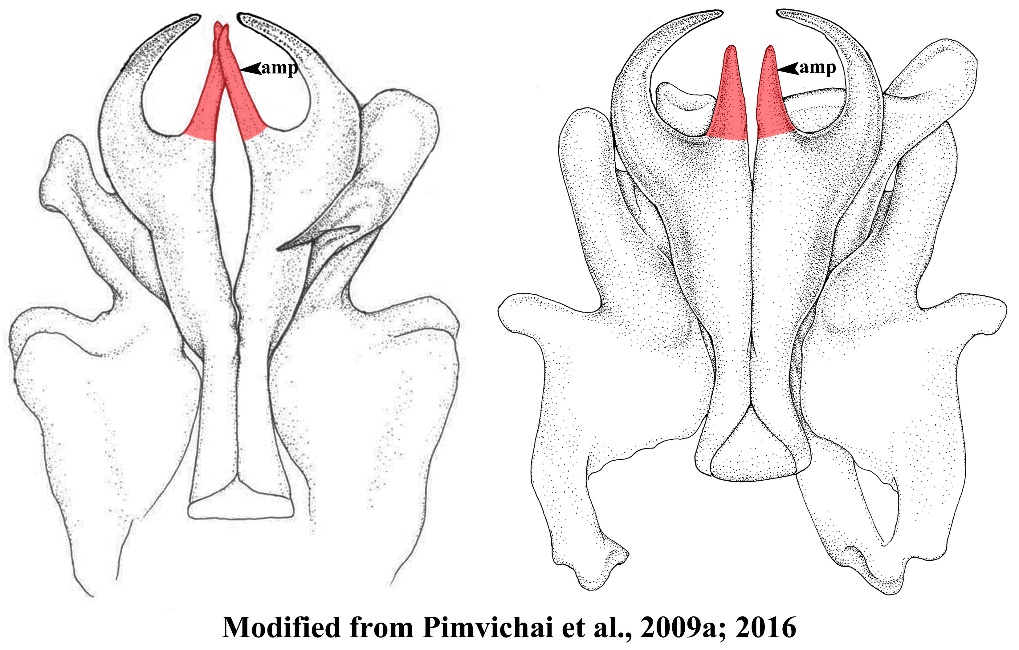 |
| --- |

17. Lateral process of anterior coxal fold (*alp*) apically without a crest; telopodite distally with a rounded lobe (*lo*); margins of spatulate lobe (*sl*) terminally meeting in a distinct angle ...................................................................................................................................***T. bispinus***

− Lateral process of anterior coxal fold (*alp*) apically with a crest…………….…….…….….18

| 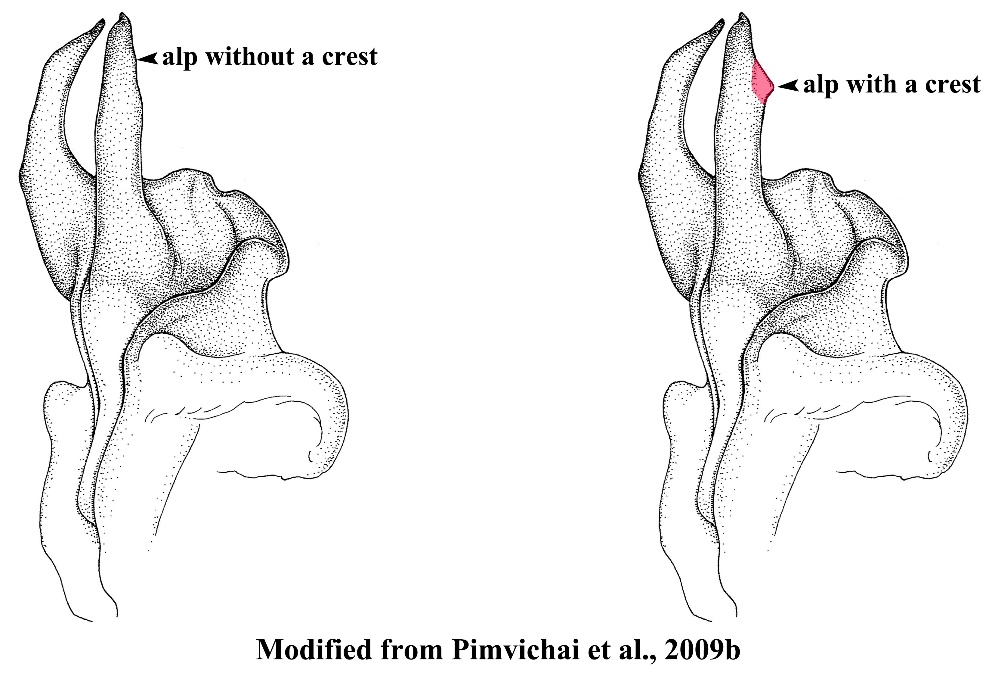 |
| --- |

18. Mesal process of anterior coxal fold (*amp*) apically irregularly tuberculate; telopodite distally without a rounded lobe (*lo*)........................................................................................***T. inflexus***

− Mesal process of anterior coxal fold (*amp*) slender, straight, its tip pointed, its mesal margin forming a strong longitudinal crest (*lc*) in posterior view…………...….……***T. mesocristatus***

19. Anterior coxal fold (*ac*) with an additional spine-like process (*aip*) between *alp* and *amp*; lateral process of anterior coxal fold (*alp*) broad, mesal margin concave, tip with serrate margins, chicken comb-like; mesal process of anterior coxal fold (*amp*) much shorter than lateral process (*alp*), directed mesodistad, simple, pointed; both femoral spines (*fe*) slender, long.........................................................................................................................***T. cristagalli***

− Anterior coxal fold (*ac*) without an additional spine-like process (*aip*) between *alp* and *amp*……………………………………………………...…………………….………….......20

| 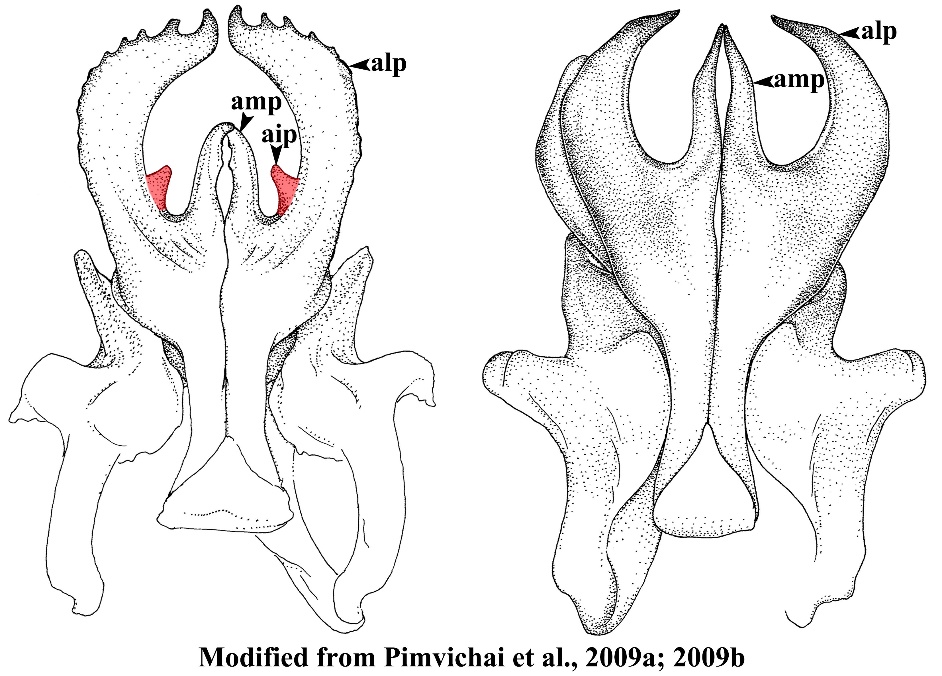 |
| --- |

20. Lateral process of anterior coxal fold (*alp*) apically without a crest, flattened, slightly curved, its laterodistal margin coarsely dentate, terminating in a short, sharp, pointed spine; mesal process (*amp*) much shorter than *alp*, directed distad, tip curving mesad, pointed; both femoral spines (*fe 1, fe 2*) long, curving backward; tibial spine (*ti*) long, not curving in horizontal plane……………………………………………………………...……...…***T. culter***

− Lateral process of anterior coxal fold (*alp*) apically with a crest extending caudad………...21

21. Lateral process (*alp*) flattened, curving mesad, laterodistal margin coarsely dentate, terminating in a short spine, tip curving against the tip of opposite side; mesal process (*amp*) much shorter than *alp*, slender, curving mesad; both femoral spines (*fe 1, fe 2*) broad, long; tibial spine (*ti*) long, curving in horizontal plane, not ending in a sharp spine…...***T. undulatus***

− Lateral process (*alp*) regularly curved, terminating in a sharp, slightly upward pointing spine; mesal process (*amp*) slightly shorter than *alp*, flattend, straight, directed distad; tibial spine (*ti*) flattend, short, curving mesad…………………………………..……………***T. planispina***

22. Telopodite with a single femoral spine…………...………………………...…..……………23

− Telopodite with two femoral spines……………………………………….........……………25

23. Lateral process of anterior coxal fold (*alp*) without an apical crest; mesal process of anterior coxal fold (*amp*) shorter than and as broad as *alp*, directed distad; femoral spine (*fe*) very long and slender.......................................................................................................***T. casjeekeli***

− Lateral process of anterior coxal fold (*alp*), with a sharp crest on the posterior surface near the tip………………………………………………………………..……………...………..24

24. Lateral process of anterior coxal fold (*alp*) flattened, slightly curved, inflexed; femoral spine (*fe*) very long, slender, with an additional lamella at base…………..…..…….***T. quadricuspis***

− Lateral process of anterior coxal fold (*alp*) regularly curved, basally broad, gradually tapering towards end and ending in sharp point; femoral spine (*fe*) very long, slender, without an additional lamella at base….........................................................................................…***T. cimi***

25. Lateral process of anterior coxal fold (*alp*) flatten, broad..……………….………………....26

− Lateral process of anterior coxal fold (*alp*) slender, regularly curved, sickle-shaped….…....27

26. Lateral process of anterior coxal fold (*alp*) terminating in a very short external spine and a very long internal one; mesal process of anterior coxal fold (*amp*) as long as *alp*; first femoral spine (*fe 1*) very short, pointed; second femoral spine (*fe 2*) very long, as long as tibial spine (*ti*); an additional lamella at both side of base of *fe 2*.*...*..............................***T. richardhoffmani***

− Lateral process (*alp*) flattened, apically curved laterad as a short spine, lateral margin of *alp* slightly folded; mesal process (*amp*) shorter than *alp*, slender, straight, directed distad, pointed; the first femoral spine (*fe 1*) very short, directed upward, situated above *fe 2*, the second *fe* (*fe2*) very long, slender, curved downward……………………...………….***T. ursus***

27. Mesal margin of lateral process of anterior coxal fold (*alp*) simple, without a caudad spine or crest; mesal process of anterior coxal fold (*amp*) much shorter than lateral process (*alp*), curved, pointed…………………………………...……….…………..……..….....*.****T. enghoffi***

− Mesal margin of lateral process of anterior coxal fold (*alp*) with a caudad small spine or crest..........................................................................................................................................28

28. Mesal margin of lateral process of anterior coxal fold (*alp*) with a small caudad crest; mesal process of anterior coxal fold (*amp*) slightly shorter than *alp*, slightly sigmoid, pointed*………………………………………………………………………………………..****T. bifurcus***

− Mesal margin of lateral process of anterior coxal fold (*alp*) with a short curved caudad spine; mesal process of anterior coxal fold (*amp*) as long as *alp*, straight……..……..***T. demangei***
